# Current Status and Potential of RNA Interference for the Management of Tomato Spotted Wilt Virus and Thrips Vectors

**DOI:** 10.3390/pathogens10030320

**Published:** 2021-03-09

**Authors:** Alexander Nilon, Karl Robinson, Hanu R. Pappu, Neena Mitter

**Affiliations:** 1 Centre for Horticultural Sciences, Queensland Alliance for Agriculture and Food Innovation, The University of Queensland, St Lucia, QLD 4067, Australia; a.nilon@uq.net.au (A.N.); k.robinson2@uq.edu.au (K.R.); 2 Department of Plant Pathology, Washington State University, Pullman, WA 99164-6430, USA; hrp@wsu.edu

**Keywords:** tomato spotted wilt virus, RNA interference, thrips, disease management, tospoviruses, double-stranded RNA, western flower thrips, *Frankliniella occidentalis*

## Abstract

Tomato spotted wilt virus (TSWV) is the type member of the genus *Orthotospovirus* in the family *Tospoviridae* and order Bunyavirales. TSWV, transmitted by several species of thrips, causes significant disease losses to agronomic and horticultural crops worldwide, impacting both the yield and quality of the produce. Management strategies include growing virus-resistant cultivars, cultural practices, and managing thrips vectors through pesticide application. However, numerous studies have reported that TSWV isolates can overcome host-plant resistance, while thrips are developing resistance to pesticides that were once effective. RNA interference (RNAi) offers a means of host defence by using double-stranded (ds) RNA to initiate gene silencing against invading viruses. However, adoption of this approach requires production and use of transgenic plants and thus limits the practical application of RNAi against TSWV and other viruses. To fully utilize the potential of RNAi for virus management at the field level, new and novel approaches are needed. In this review, we summarize RNAi and highlight the potential of topical or exogenous application of RNAi triggers for managing TSWV and thrips vectors.

## 1. Introduction

Among the most economically important plant viruses, tomato spotted wilt virus (TSWV) is considered the second most significant virus species in global agricultural industries [1]. TSWV has a wide host range and is capable of infecting more than 1500 plant species, which includes important agronomic and horticultural crops and numerous weeds [2]. Symptoms of TSWV are highly variable and depend on the host plant, virus isolate, and environmental factors such as temperature. Common symptoms include chlorosis, necrotic lesions, ring spots, mottling, stunting, and death of young plants. While early infection can lead to total crop loss, more common outcomes of the infection include a significant reduction in produce quality and marketable yield [3,4].

## 2. Thrips as a Vector of TSWV

The reason for the prevalence and significance of TSWV as a disease in horticultural crops is its insect vector, thrips. Thrips feed on plant tissue utilising a “punch-and-suck” method [5]. Briefly, the insect uses its mandible to puncture plant tissue cells, and then ingests the cellular fluid using its maxillary stylet [6]. This feeding activity involves the release of saliva from the salivary glands via the stylet tube; this is critical to thrips’ function as a viral vector. TSWV infects and resides persistently within the thrips’ salivary glands, so that virus particles are inoculated into the host plant through the saliva emitted during feeding [7]. Plant inoculation and infection occurs in as little as five minutes of feeding for *Frankliniella occidentalis* [8], while individual feeding events by thrips can extend for as long as thirty minutes, providing ample time for infection to occur [6,9]. Interestingly, thrips only become competent and persistent vectors of tospoviruses when they feed upon infected plant tissue as instar larvae [10]. Adults can acquire tospoviruses through such feeding, but will be incapable of transmitting TSWV [4,11,12].

## 3. Current Management Strategies

Considering that TSWV and its thrips vectors have wide and overlapping host ranges, an integrated disease management approach has to be used to manage TSWV and other thrips-transmitted viruses [11,12]. Because TSWV is transmitted persistently by thrips [13], suppression of this disease is focused on managing thrips as both a crop pest as well as a virus vector; fewer thrips means less spread of TSWV [14,15]. Thrips have shown increasing resistance to several broad-spectrum insecticides [16], and while the effectiveness of selective pesticides varies with thrips species [17], Spinosad is commonly cited as an effective choice against *F. occidentalis* (western flower thrips), the most prolific vector of TSWV [18]. However, thrips populations can rapidly develop pesticide resistance [19], especially when placed under the pressure of overuse and a lack of insecticide rotation. Thrips also tend to evade the application of contact pesticides, due to their small size and habit of hiding from surface sprays [20]. This has increased the need for the use of cultural practices to supplement chemical treatments in reducing thrips populations.

Cultural practices for TSWV management largely consist of methods to remove virus reservoir plants outside the crop field to prevent the movement of vector thrips from reservoirs into crops. These strategies can include outright disposal of green bridges for TSWV [21], planting crops a significant distance away from potential reservoirs to limit the risk of thrips movement [22], the use of selective planting times based on thrips reproductive cycles relative to the vulnerable life stages of plants [23], and using UV-reflective mulches to reduce the landing of airborne thrips on crops by interfering with their perception [24]. A risk index for TSWV infection in peanut crops has been developed and updated annually, called Peanut Rx. This risk calculator takes into account a wide variety of factors, such as the peanut cultivar, crop plant density, insecticide use, and planting dates, in order to provide a weighted estimation of the risk posed by TSWV under the given conditions [25]. This allows growers to make informed decisions about cropping strategies to minimise the potential for TSWV outbreaks. The south-eastern states of the United States of America have been home to extensive efforts to develop and improve integrated pest management strategies for TSWV in peanut since the 1980s, considering chemical and cultural practises for thrips control, as well as the use of resistant plant species [26].

There has also been significant efforts to produce varieties of tomato plants that are resistant to thrips, with *Lycopersicon hirsutum, L. hirsutum f glabratum*, and *L. pennellii* being poor hosts to thrips nymphs, preventing feeding and subsequent infection with TSWV [27,28]. However, even resistant cultivars have limitations, with numerous reports of resistance-breaking TSWV, demonstrating that this virus can overcome various forms of plant-based resistance, such as the Sw-5 gene in tomatoes [29,30,31] and the Tsw gene in capsicum [32,33]. As with pesticide use, resistant cultivars are not a foolproof solution due to the potential of TSWV to overcome such resistances, necessitating the use of multiple management strategies.

Biological control strategies aim to reduce the populations of insect pests through the use of natural predators [34]. Studies have demonstrated that several members of the *Orius* genus (commonly known as minute pirate bugs), such as *O. niger*, *O. armatus*, and *O. laevigatus*, can significantly reduce the thrips populations when introduced under glasshouse conditions and in seedling nurseries [35]. However, there are limited studies that directly investigate how these predators affect TSWV incidence, with data only on their effects on thrips numbers, and these insects are often vulnerable to the same insecticides used to control thrips [36].

## 4. RNA Interference and Its Role in Plant–Virus Interactions

**Figure 1:**
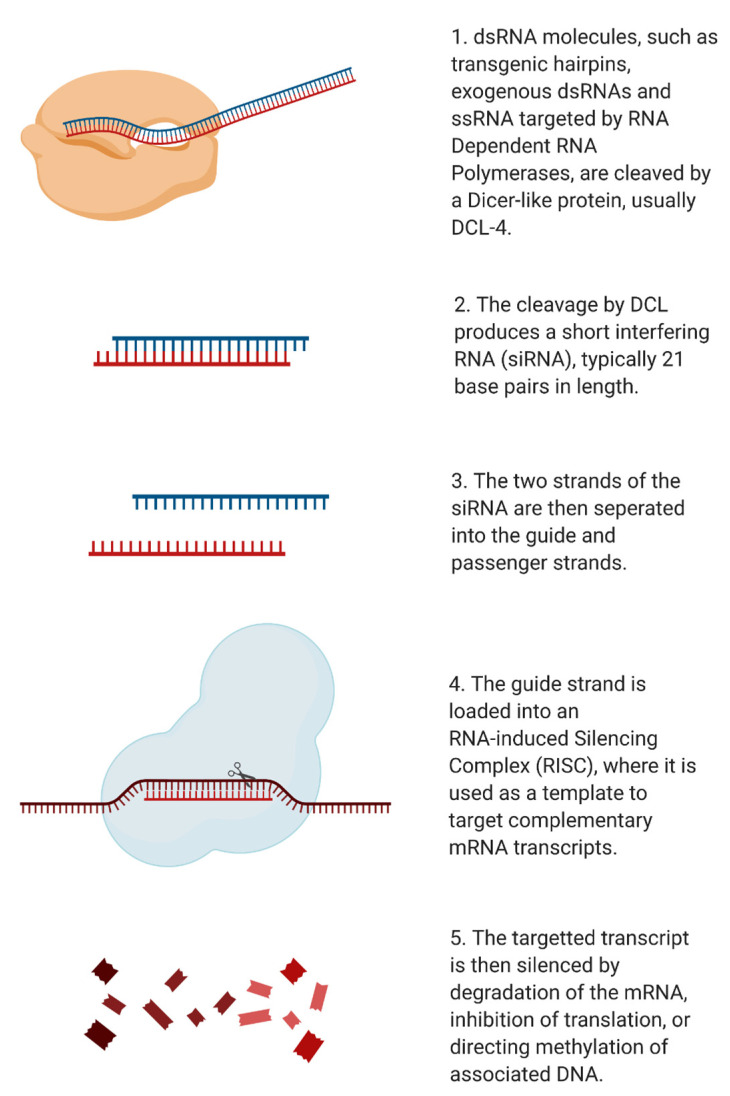
Biogenesis of siRNA molecules in plants for silencing of complementary mRNA. dsRNA serves as a substrate for producing siRNAs, which function as a template to silence matching mRNA molecules. This RNAi pathway serves an important function in genetic regulation and, in the case of plants, immune response. Created with BioRender.com.

RNA interference (RNAi) is a mechanism of genetic regulation observed across plants, animals, and fungi that utilises small RNA (sRNA) as trigger molecules for the manipulation of gene expression. sRNAs are derived from double-stranded RNA (dsRNA) and come in a variety of forms, each differing in structure, function, and biogenesis. For example, micro RNA (miRNA, ~22 nucleotides long) are transcribed from plant genomes to regulate expression of endogenous genes [37], while small interfering RNA (siRNA, 20–25 nucleotides long) are derived from exogenous dsRNA sequences or the products of miRNA-directed silencing [38]. The biogenesis of siRNAs is illustrated in Figure 1.

The mechanisms of RNAi have been well-studied and several reviews have been published on the topic [39,40]. The RNAi pathway influences numerous elements of an organism’s biology, including development [41], transposon silencing [42], and viral defence [43,44]. Plants utilise RNAi against invading pathogens using viral siRNAs (vsiRNAs), which silence expression of critical virus genes to impede infection. vsiRNAs can be generated in several ways, although there is debate about the significance and likelihood of each method. Viruses with singled-stranded RNA (ssRNA) genomes, such as tospoviruses, demonstrate double-stranded intermediates during replication due to the action of their RNA-dependent RNA polymerase (RdRp), and these dsRNA forms are potential substrates to generate vsiRNAs [45]. Molnár et al. [46] and Pantaleo et al. [47] further suggest that specific regions of a viral genome may be more heavily targeted for vsiRNA generation due to native secondary stem-loop structures, which result in spans of double-stranded RNA. It is most likely that secondary structures within viral genomes are the main progenitors of vsiRNA, rather than dsRNA replicative intermediates. In the case of TSWV infection, it has been found that vsiRNAs target the intergenic regions of the TSWV’s genome less than the ORFs, and that the presence or absence of the silencing suppressor protein (NSs) from TSWV influences targeting of the viral genome with vsiRNAs, suggesting viral factors are able to modify, reduce, or subvert the plant cells’ sRNA biogenesis machinery [48,49].

## 5. RNAi and TSWV

Viral genomes often encode a silencing suppressor to repress or subvert plant RNAi functions to prevent their own inhibition. Silencing suppressors are a common theme across plant viruses, and utilise many different mechanisms to counter host antiviral RNAi responses [50] as well as serving as pathogenicity determinants, allowing a virus to overcome natural host defences [51].

The NSs protein of tospoviruses was shown to be the silencing suppressor [52], and functions to sequester siRNA molecules to prevent their loading onto the RISC, rendering them incapable of targeting viral transcripts. This RNA-binding function is related to two conserved domains within the NSs gene, which have been determined to be essential for the NSs function [53], although efforts to investigate the significance of the NSs protein structure in this regard have been impeded by difficulties in conclusively predicting its folded structure [54]. Notably, NSs is capable of binding to all manners of sRNAs, including endogenous miRNAs, as well as large dsRNA precursors, directly inhibiting their processing to siRNAs [55].

The full extent of the NSs’ function to suppress silencing in a host plant is presently unclear. A study that utilised the NSs protein alongside viruses deficient in their own silencing suppressors concluded that while NSs did not appear to prevent the initial biogenesis of vsiRNAs or their systemic spread through the plant, the reduced accumulation of vsiRNAs compared to controls suggested that the suppressor rather interferes with the amplification of those trigger molecules [56]. This may relate to NSs’ ability to bind to long dsRNAs, which could otherwise serve as substrates for siRNA biogenesis, although the protein supposedly has a greater binding affinity for siRNAs [55]. Using NSs to infiltrate individual leaves of a plant, simultaneously expressing and silencing green fluorescent protein (GFP), demonstrated a quantifiable reduction in systemic GFP silencing in a dose-dependent manner [57]. The discrepancy regarding NSs’ impact on systemic silencing may be due to the different requirements for suppression of transgene silencing and antiviral silencing [45], as proposed by Ocampo et al. [56]. Regardless, NSs serves to sequester key RNAi trigger molecules.

Systemic expression of the TSWV’s NSs protein in capsicum plants in comparison to the virus’s other non-structural proteins demonstrated that this silencing suppressor is responsible for attracting thrips to TSWV-infected plants through suppression of terpene synthesis, which would otherwise assist in repelling these insects [58]. NSs also serves as a recognition factor for a hypersensitive response in TSWV-resistant crop species utilising the *Tsw* resistance gene [59,60], with mutations to the NSs sequence resulting in resistance-breaking isolates of the virus in as little as a single amino acid change [32,61]. Investigations into the N-terminal domain of NSs demonstrated mutations within this region could result in both loss of avirulence recognition for *Tsw* and suppression activity at once, although those functions were supposedly not inextricably linked [62]. Finally, NSs also appears to have an essential function in the vector thrips, with a defective silencing suppressor impeding virus accumulation in adult thrips; it is possible that NSs also serves as a suppressor of RNAi in insects, a function that may be necessary to accomplish persistent infection within thrips for transmission [63].

## 6. Transgenic RNAi for Induced Viral Resistance

While plant viruses are typically able to overcome RNAi defences in their hosts using silencing suppressors, transgenic RNAi defences have nonetheless demonstrated significant value in creating plants with resistance to specific viruses. The early years of RNAi research are full of examples where researchers introduced viral transgenes into plants, often expressing coat protein sequences, and observed delayed symptom development or outright resistance [64,65,66]. Typically, transgenic strategies involve engineering an expression cassette containing a sequence homologous to the desired viral gene to be targeted [67,68]. The sequence is present in both the sense and antisense forms, with the inverted repeats being linked by a spacer sequence (usually an intron sequence to improve downstream processing efficiency) [69]. When transformed into a host plant and expressed as ssRNA, a long hairpin RNA is transcribed, with the sense and anti-sense sequences annealing together to form dsRNA, while the spacer functions as a hinge loop to connect them together. This dsRNA serves as a substrate for the generation of siRNAs to target the matching viral sequences.

The first published study that used transgenic RNAi to create TSWV resistance did so inadvertently; Gielen et al. [70] transformed tobacco plants to express TSWV’s nucleocapsid (N) protein, believing that high titres of this protein during infection causes it to function in a “replicative” role that would actually inhibit TSWV infection during its early stages. While their transgenic plants did indeed demonstrate resistance to TSWV, it was only later defined that this resistance was through the action of RNA homologous to the N gene sequence, not the N protein itself, given that translation of the mRNA was not necessary to achieve resistance [71]. Early working theories in such studies proposed that the viral resistance was mediated by expression of viral proteins [72], and focus only shifted to the action of RNA [73,74] and then to dsRNA [75] with subsequent investigations.

Thereafter, the use of RNAi to induce resistance against TSWV has been thoroughly investigated. A study that produced transgenic plants expressing RNA targeting other genes within the TSWV genome for silencing found that significant resistance was only achieved when RNA matching the non-structural movement protein (NSm) or N genes were expressed, suggesting that the RNAi response is sequence specific, and that not all genes are equally as effective as RNAi targets [76]. Similarly, it was found that a minimum sequence length was necessary to trigger RNAi against the N gene, with constructs containing <59 bp failing to provide virus resistance, while sequences of 110 bp or more were effective [77]. Effective sequence lengths highlight the hurdles involved in initiating RNAi in planta, since not only is the silencing target critical, but so is the form of the triggering dsRNA molecule. Notably, an attempt to express sequences to silence the NSm and NSs genes in *N. benthamiana* infected with TSWV resulted in limited viral protection in comparison to results targeting the N gene [78]. A similar conclusion was reached when using artificial miRNAs with sequences homologous to the N and NSs genes [79]; in short, it appears as though the N gene is the best target for RNAi to achieve protection against TSWV.

Transgenic RNAi strategies can be used to create plants resistant to multiple viruses. Developing chimeric constructs containing an N gene sequence from TSWV and three other tospoviruses species (*Groundnut ringspot virus*, *Tomato chlorotic spot virus*, and *Watermelon silver mottle virus*) resulted in resistance to all of these viruses, as plants were able to produce siRNAs matching each virus from a single dsRNA substrate [80]. Furthermore, combining sequences from the TSWV’s N gene and the C2 gene of tomato leaf curl Taiwan virus, from the *Begomovirus* genus, provided cross-protective resistance to both viruses [81]. Similar cross-protective effects have been induced with a single sequence from the *Watermelon silver mottle virus* genome, providing resistance to several other tospoviruses due to sufficient conservation exhibited across tospoviruses in the targeted sequences [82]. However, that study targeted the RNA-dependent RNA polymerase (RdRp) gene of the virus, rather than the more typical N gene. In this way, the use of chimeric constructs or the targeting of conserved sequences makes it possible to produce transgenic plants with cross protection beyond the scope of TSWV.

## 7. RNAi in Thrips

RNAi functions to some degree in most insects, with the majority of work being performed in and inferred from model insects such as *Drosophila melanogaster* [83]. Insects utilise the same mechanistic pathways to achieve RNAi as previously described [84], wherein dsRNA functions as a substrate leading to the silencing of homologous sequences. Notably, as in plants, RNAi plays a role in viral defence for insects [85]. Insect RNAi machinery can utilize viral genome replicate intermediates and secondary structures for siRNA biogenesis [86], but are also able to reverse-transcribe templates such as defective viral genomes into DNA to contribute to siRNA production [87,88]. However, there are additional hurdles to RNAi responses in insects that should be considered. For example, the RdRps that propagate RNAi signals are absent in insects and the RNA uptake mechanisms are largely undefined, yet the spread of silencing signals and systemic RNAi have been observed, leaving the potential mechanisms behind the amplification and propagation unclear [89,90]. Furthermore, the effectiveness of RNAi between insect species and in different tissues or life stages of a particular species are highly variable [91,92]. As a result, applications and protocols for RNAi in one species may not necessarily transfer to another.

Nonetheless, studies have demonstrated that introducing dsRNA into insects through means such as microinjection [93] or feeding [94] can result in a significant reduction in target gene expression. It can be assumed that because these studies have shown a potentially functional RNAi mechanism in pest insect species, RNAi holds the potential to be used as a bio-insecticide by silencing critical genes, increasing susceptibility to traditional insecticides, directly causing mortality, or otherwise being deleterious to the pest [95]. Due to the impracticality of injecting pests with dsRNA outside of a research context, RNAi is typically employed by using transgenic plants constitutively expressing a dsRNA sequence against the targeted insect gene [95,96]. Here, insects feed on the plant tissue to take up the dsRNA, and then, in theory, will initiate an RNAi response once the dsRNA spreads from the midgut [89]. This may be a potential avenue to control thrips and TSWV.

There has been a limited amount of research on thrips and RNAi. Badillo-Vargas et al. [97] reported a significant decrease in survival and fertility by introducing dsRNA into *F. occidentalis* by microinjection, targeting the transcript of the *V-ATPase-B* gene, a vacuolar ATP synthase involved in solute transport across plasma membranes as well as lumen pH regulation [98]. Whitten et al. [99] also achieved success by feeding *F. occidentalis* on media containing dsRNA-expressing bacteria. The expressed dsRNA targeted an essential alpha-tubulin gene, and resulted in significant mortality of the insects, especially those in the larval life stage. More recently, Han et al. [100] developed a specialised bioassay system, in which kidney bean leaf discs were placed on solutions of in vitro synthesized dsRNA targeting an array of biologically significant genes, including *toll-like receptor 6* and *coatomer protein subunit epsilon,* within custom-made containment chambers, for passive uptake of dsRNA into the leaf tissue. Thrips nymphs (2–3 days old) were infested onto those leaf discs, still in contact with the dsRNA solution, and lethality was recorded through direct observation, as well as quantitating gene knock-down through qPCR. Their results demonstrated a significant decrease in the transcription levels of the associated genes, indicating that an RNAi response had been initiated by dsRNA uptake, and the authors were able to shortlist a number of genes that were successful in inducing mortality in the thrips through dsRNA feeding [100].

Andongma et al. [101] examined the potential of inducing RNAi in *F. occidentalis* through direct feeding on dsRNA with a vATPase-B sequence; they found that attempting to feed the thrips on dsRNA in an artificial diet solution was ineffective due to dsRNA degradation induced by the feeding process. However, feeding the insects on plants that had taken up that dsRNA by a petiole-dip method did result in significant thrips mortality, suggesting that RNAi in thrips is possible through indirect feeding methods, albeit with some caveats that may impose restrictions on the delivery method. This is a critical point, as more direct methods like microinjection and dsRNA-producing bacteria are unlikely to translate to practical outcomes as a pest management strategy for thrips.

## 8. Future Potential for RNAi against TSWV

While RNAi holds great potential to develop TSWV-resistant crop plants, there are issues with using transgenic plants to bolster RNAi-based virus resistance. Viruses are capable of overcoming RNAi sequences targeted against them, as the positive selection pressure induced by resistant plants can result in spontaneous mutations in the viral genome, reducing the homology between the siRNAs and target transcripts. This has been demonstrated in transgenic plants utilising RNAi against TSWV to silence the viral N gene, with positive pressure being exerted on specific amino acids of the sequence [102,103]. Furthermore, consumer acceptance of transgenic crops is still limited, and genetically modified food requires extensive regulation, making such plants difficult to transfer to practical applications [104,105].

Topical application of RNAi is an emerging space for crop protection, ranging from viruses to insect pests to fungi [106,107]. In these applications, either dsRNA or siRNAs are applied exogenously as triggers of RNAi without the need for integration into the plant genome through transgenic methods. Foliar application of dsRNA to induce viral resistance is rapidly becoming the popular approach to topical RNAi thanks to its ease of use. In this strategy, a dsRNA sequence is designed to target a critical gene of a given virus and is produced through methods such as in vitro synthesis [108] or bacterial expression [109]. The dsRNA is then topically applied to plant tissue, typically through mechanical co-inoculation along with the targeted virus. The exogenous dsRNA serves as a substrate for siRNA biogenesis and allows the plant to initiate an RNAi response against the targeted virus, resulting in protection against the disease. This method was first demonstrated to provide protection against pepper mild mottle virus in *N. tabacum* using in vitro synthesized dsRNA [110], and has since been used for an array of viruses, such as Cymbidium mosaic virus [111], sugarcane mosaic virus [112], and cucumber mosaic virus [113]. Recently, mechanical co-inoculation of dsRNA targeting the N gene of TSWV has been shown to provide protection in *N. benthamiana* [114]. While mechanical co-inoculation of the dsRNA is most common, some investigations have shown success through spray application of dsRNA alone, achieving an RNAi effect through passive uptake of dsRNA from the leaf surface [115].

While promising, there are limitations to the emerging strategy of topical RNAi. DsRNA on the surface of a plant is vulnerable to degradation from UV radiation and environmental nucleases, resulting in topical RNAi having a limited window of efficacy, typically just 5–7 days [109,112]. Additionally, most studies into topical RNAi for virus protection introduce dsRNA to a plant through mechanical co-inoculation with the virus, a method which is not suitable for practical application in disease management. The use of layered double hydroxide particles as a carrier for dsRNA in topical application has been shown to protect the nucleic acid against UV and nuclease degradation, resulting in effective viral protection 20 days post spray [115]. This method has also been shown to provide protection from aphid transmission of bean common mosaic virus, demonstrating that topical RNAi is effective when the virus is introduced by an insect vector, not just mechanical inoculation [116]. Using such an approach, topical RNAi has the potential to form part of integrated disease management strategy as an alternative to insecticides.

**Figure 2:**
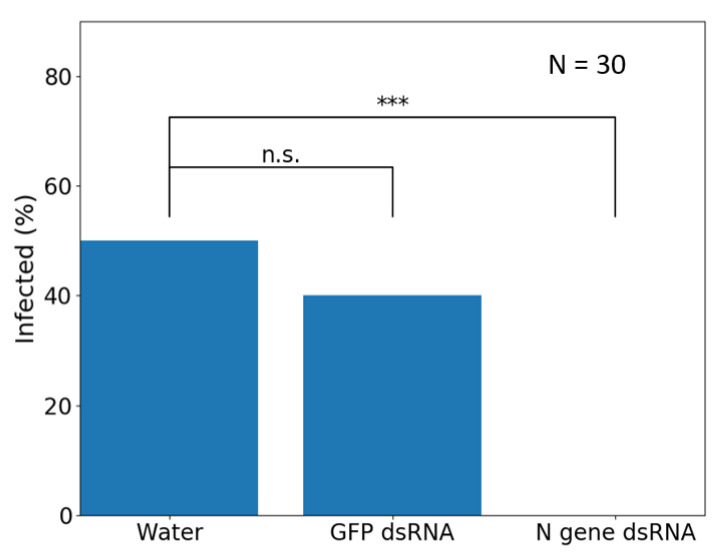
Effect of topical application of dsRNA on infection by tomato spotted wilt virus (TSWV). Capsicum plants (N = 30) were sprayed with either water, chemically synthesized GFP dsRNA (0.3 mg/mL) as an off-target negative control, or bacterially expressed dsRNA homologous to the TSWV’s N gene sequence (0.3 mg/mL). At 24 h post treatment, plants were challenged with TSWV by mechanical inoculation. Fourteen days after the viral challenge, tissue samples were collected for qualitative TSWV ELISA analysis. Fisher’s exact test (*p* = 0.05) was used to compare the infection incidence of the dsRNA treatments to the water negative control (*** = *p* < 0.001). The foliar application of N gene dsRNA has resulted in significantly reduced incidence of TSWV infection, due to the uptake of dsRNA and the resulting RNAi response against the virus. None of the plants treated with N gene dsRNA were found to be infected with TSWV. GFP dsRNA does not provide resistance to TSWV due to the sequence specificity of the response. See Supplement S2 for raw data.

As part of ongoing research for the development of novel delivery approaches to trigger RNAi in plants for crop protection, the effect of dsRNA targeting the N gene of TSWV for silencing was evaluated as a topical spray on capsicum plants (*C. annuum*). Under glasshouse conditions, TSWV-susceptible capsicum cv. Yolo Wonder plants were sprayed with solutions of water, a non-specific GFP dsRNA, or N gene dsRNA produced by bacterial expression. One day after spray application, the plants were challenged with TSWV by mechanical inoculation. Fourteen days after viral challenge, the TSWV infection incidence among the groups was determined by qualitative ELISA. Changepoint analysis [117] demonstrated a significantly reduced TSWV incidence in the plants treated with N gene dsRNA (refer to Supplement S1 for methods). This demonstrated the practical potential of topical RNAi to manage TSWV infection (Figure 2).

**Figure 3:**
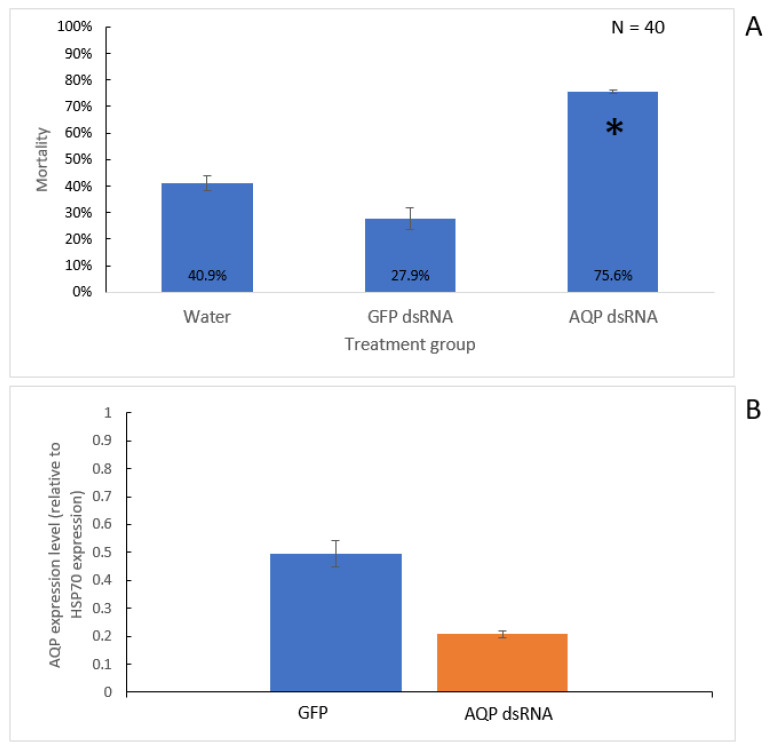
Mortality and gene expression of thrips feeding on leaf tissue treated with topically applied dsRNA. Water, in vitro synthesized GFP dsRNA, or dsRNA targeting an aquaporin (AQP) gene of *F. occidentalis* were sprayed onto capsicum leaves, supplemented by paraffin oil, which were then applied to agar plates the following day and sealed with adult *F. occidentalis* (approximately 20 per plate, two plates per treatment). Mortality was counted over 6 days, and RNA was extracted from the surviving thrips for expression analysis by qPCR. (**A**) Mortality in treatment groups were compared by pooling numbers across the duplicates and performing Fisher’s exact test (*p* = 0.05), comparing the AQP dsRNA treatment to the water and GFP controls, with a Holm–Bonferroni correction. An asterisk (*) indicates this test found that mortality in the AQP treatment was significantly greater than both controls. The graph shows the average mortality rate across the duplicate trials, with error bars showing the standard deviation. (**B**) Average expression of the targeted aquaporin gene across the duplicated trials was quantitated relative (∆∆Cq method) to a heat shock protein 70 (HSP70) gene in both the GFP and AQP dsRNA treatment groups. Error bars indicate the standard deviation across duplicate trials. A trend towards decreased AQP expression in the AQP dsRNA treatment group was observed compared to the GFP dsRNA treatment. See Supplement S2 for raw data.

In addition, we have also conducted studies targeting thrips using exogenous application of dsRNA to further manage TSWV by directly reducing the populations of thrips vectors. As previously discussed, studies have successfully demonstrated the potential to utilise dsRNA in inducing mortality in thrips through feeding on plant tissue soaked in dsRNA solutions [97,100]. There have also already been extensive efforts to explore the possibility of translating RNAi research into practical management of other pests, most notably the Colorado potato beetle [118,119,120]. We extended the topical application of dsRNA to plant tissue as a spray solution to trigger RNAi to thrips (refer to Supplement S1 for methods). Capsicum leaves were treated with sprays of water, GFP dsRNA, or dsRNA targeting an *aquaporin* homolog gene in *F. occidentalis*, previously shown to be effective in inducing lethality through RNAi [100]. One day after spray application, the leaves were detached and placed onto agar media plates in duplicate along with approximately 20 *F. occidentalis* adults. Over six days, mortality was determined by direct observation before extracting total RNA to determine the expression change of the targeted *aquaporin* gene. A trend towards increased mortality was observed in thrips feeding on the *aquaporin* dsRNA-treated leaves compared to the controls, accompanied by a reduction in relative gene expression (Figure 3).

Further consideration in the deployment of RNAi is the risk of off-target effects. RNAi mechanisms are present across plant species, as well as insects and animals. While it is easy enough to generate a dsRNA sequence for homology against a targeted sequence, it is a significantly greater hurdle to ensure the dsRNA sequence does not possess sufficient homology against unintended gene targets, and thus be toxic to unintended organisms [121,122]. To limit the risk of off-target effects, it is necessary to consider other organisms that may come into contact with topically applied dsRNA (particularly beneficial insects such as pollinating bees and butterflies) and whether the dsRNA could trigger an RNAi response in those organisms. It may become necessary to alter the selected dsRNA sequence for topical RNAi to reduce the risk of off-target effects, with a particular focus on the 21 nt stretches that produce effective siRNAs [94].

## 9. Conclusions

TSWV continues to be a serious threat to agriculture, given its ability to break host resistance and the capability of its thrips vectors to develop resistance to traditional pesticides. For this reason, there is a need to explore novel and effective strategies to introduce resistance to TSWV and its thrips vectors in crop plants. Researchers from around the world are developing new pest management options that are totally different formulations to what we currently think of as pesticides. In laboratory and glasshouse trials, topical or exogenous application of RNAi is showing some very attractive properties compared to traditional pesticides, including zero reduced crop residues, minimal off-target impacts, and low risk for resistance. Furthermore, a body of evidence is being generated addressing the environmental stability of RNAi and regulatory paradigms for its wider application. Presently, the cost of producing dsRNA, as the RNAi-triggering, biologically active agent, is being researched to ascertain its economic feasibility. Another area that is gaining momentum is novel carriers of RNAi triggers to make it suitable for field applications. The progress made so far in developing novel and effective delivery methods for exogenous introduction of dsRNA could be a new addition to the existing toolbox for managing TSWV and thrips vectors.

## Data Availability

The data presented in this study are available in Supplement S2.

## Supplementary Materials

The following are available online at https://www.mdpi.com/2076-0817/10/3/320/s1, Supplement S1 contains additional details on the methods used for the preliminary results presented here, Supplement S2 contains the raw data presented in Figure 2 and Figure 3.
