# Supplementary material for: Current Status and Potential of RNA Interference for the Management of Tomato Spotted Wilt Virus and Thrips Vectors
Source: Pathogens. 2021 Mar 9;10(3):320. doi: 10.3390/pathogens10030320 (PMC8001667; doi:10.3390/pathogens10030320)
Supplement: Supplementary file 1 [file pathogens-10-00320-s001.zip › 210303 - Supplement S1.docx]

Topical RNAi for TSWV

*E. coli* (HT115) (sourced from the Caenorhabditis Genetics Center) was transformed with a L4440 vector containing an expression cassette made up of 300bp TSWV N-gene inverted repeats flanking a 466 bp tobacco peroxidase intron sequence to serve as a hairpin loop (accession D11396). The resulting *E. coli* was named HT115-Ngene. The HT115-Ngene strain was used to express TSWV Ngene dsRNA, following established protocol for HT115 expression [1] using TB media for expression.

*Capsicum annuum* was used as a model species for testing topical RNAi against TSWV infection, using the Yolo Wonder variety for its viral susceptibility, at the 4-6 true leaf stage of growth. Plants were kept under glasshouse conditions (maintained at 27°C during daylight hours, and 20°C at night, following natural light cycles).

dsRNA spray treatments were prepared at a concentration of 0.3 mg/mL in sterile water. Control treatments consisted of water and an off-target dsRNA (Genolution, South Korea) using a green fluorescent protein (GFP) sequence. DsRNA sprays were applied to 30 capsicum plants and plants were maintained under glasshouse conditions, as outlined above. Approximately 24 hours post spray, capsicum plants were challenged with TSWV by mechanical inoculation [2]. The infection incidence was determined by ELISA 14 days post viral challenge (Agdia, cat#SRA 39300/0500).

Topical RNAi for thrips

Thrips culturing was based on a previously published protocol [3]. *Frankliniella occidentalis* (Western flower thrips, or WFT) adults were collected from a colony maintained at the University of Queensland. An aquaporin (AQP) transcript (accession XM_026423864.1) was selected as a potential gene target for lethal RNAi in *F. occidentalis* based on previous studies demonstrating success when silencing this gene [4,5]. Chimeric primers incorporating 5’ and 3’ T7 polymerase recognition sequences were designed for amplification of the AQP transcript from *F. occidentalis* cDNA [6]. Total RNA was extracted from colony thrips, treated with DNAse (Thermo Fisher Scientific, cat#AM1907) and cDNA synthesized using a SensiFAST cDNA Synthesis kit (Bioline, cat#BIO-65054). Linear DNA templates were amplified by PCR using Phusion High-Fidelity DNA Polymerase (New England BioLabs, cat# M0530S), along with the above designed primers. dsRNA was synthesized using a HiScribe T7 High Yield RNA Synthesis Kit (New England BioLabs, cat#E2040S), following standard protocol.

Two leaves on capsicum plants (Yolo Wonder variety) were marked and sprayed with treatments of water, GFP dsRNA or AQP dsRNA, at 0.3 mg/mL. Treatments were allowed to dry on leaves before being detached and placed onto solid media plates (1% agar, 20 µg/mL benzimidazole). Approximately 15 early adult WFT were place on each leaf and living thrips were counted by observation at various time intervals.

Following the ~96 and ~144-hour timepoints, active thrips were collected from one plate, pooling individuals within treatments at each timepoint. The assay was performed in duplicate. Total RNA was extracted and cDNA synthesized as above. qPCR was performed to assess respective reduction in AQP transcript abundance using the SensiFAST SYBR No-ROX Kit and qPCR primers designed to target AQP transcript as well as two reference genes comparators, heat shock protein 70 (HSP70) and heat shock protein 60 (HSP60).

Each sample was assayed in triplicate, including no template and no reverse transcriptase controls, using all three primer sets (HSP70, HSP60 and AQP). The ∆∆Cq values [7] of the target genes were determined in the GFP dsRNA control group and the AQP dsRNA treatment group, using the water control group as a reference standard. Simple analysis of the duplicate assay was performed based on the mean ∆∆Cq values in comparison to the GFP control group, considering trends based on the standard deviation of the mean.

Reference

1. Senthil-Kumar, M.; Mysore, K.S. Caveat of RNAi in Plants: The Off-Target Effect. In *RNAi and Plant Gene Function Analysis: Methods and Protocols*; Kodama, H., Komamine, A., Eds.; Methods in Molecular Biology; Humana Press: Totowa, NJ, USA, 2011; pp. 13–25, ISBN 978-1-61779-123-9.
2. Fletcher, S.J.; Reeves, P.T.; Hoang, B.T.; Mitter, N. A Perspective on RNAi-Based Biopesticides. *Front. Plant Sci.* **2020**, *11*, doi:10.3389/fpls.2020.00051.
3. Mandal, B.; Pappu, H.R.; Culbreath, A.K. Factors Affecting Mechanical Transmission of Tomato Spotted Wilt Virus to Pea nut (Arachis Hypogaea). *Plant Dis.* **2001**, *85*, 1259–1263, doi:10.1094/PDIS.2001.85.12.1259.
4. DeGraaf, H.E.; Wood, G.M. An Improved Method for Rearing Western Flower Thrips Frankliniella Occidentalis. *Fla. Entomol.* **2009**, *92*, 664–666.
5. Singh, S.; Gupta, M.; Pandher, S.; Kaur, G.; Goel, N.; Rathore, P.; Palli, S.R. RNA Sequencing, Selection of Reference Genes and Demonstration of Feeding RNAi in Thrips Tabaci (Lind.) (Thysanoptera: Thripidae). *BMC Mol. Biol* **2019**, *20*, 6, doi:10.1186/s12867-019-0123-1.
6. Untergasser, A.; Cutcutache, I.; Koressaar, T.; Ye, J.; Faircloth, B.C.; Remm, M.; Rozen, S.G. Primer3—New Capabilities and Interfaces. *Nucleic Acids Res.* **2012**, *40*, e115, doi:10.1093/nar/gks596.
7. Livak, K.J.; Schmittgen, T.D. Analysis of Relative Gene Expression Data Using Real-Time Quantitative PCR and the 2−ΔΔCT Method. *Methods* **2001**, *25*, 402–408, doi:10.1006/meth.2001.1262.
